# Intratumor heterogeneity of lymphoma identified by multiregion sequencing of autopsy samples

**DOI:** 10.1111/cas.15178

**Published:** 2021-11-21

**Authors:** Kenichi Makishima, Yasuhito Suehara, Yoshiaki Abe, Keiichiro Hattori, Manabu Kusakabe, Ryota Matsuoka, Shigeru Chiba, Mamiko Sakata‐Yanagimoto

**Affiliations:** ^1^ Department of Hematology, Graduate School of Comprehensive Human Sciences University of Tsukuba Tsukuba Japan; ^2^ Department of Hematology University of Tsukuba Hospital Tsukuba Japan; ^3^ Department of Hematology, Faculty of Medicine University of Tsukuba Tsukuba Japan; ^4^ Department of Pathology, Faculty of Medicine University of Tsukuba Tsukuba Japan

## CONFLICT OF INTEREST

The authors declare no conflicts of interest.

Dear Editor,

Lymphomas occur at either secondary lymphatic organs, especially lymph nodes, or at extranodal organs. Once lymphoma cells arise in one site, they are believed to spread through the vascular network, even when lesions are not clinically observed by imaging. We performed multiregion sequencing of 12 lymphoma samples from a patient and constructed a phylogenetic tree. This study revealed a dynamic tumor evolution and intratumor heterogeneity of lymphoma.

Recent progress in next‐generation sequencing technologies has shown that tumor cells in many solid cancers undergo dynamic clonal evolution.^1^ Notably, diverse clones have been observed in biopsy samples acquired from primary or metastatic legions in solid cancers.^2^, ^3^, ^4^ In contrast, intratumor heterogeneity (ITH) in lymphomas is less characterized: samples are taken only for diagnosis and multiregional sample collection is rarely performed in living patients, because chemotherapy rather than surgery is the standard treatment for most lymphoma subtypes. One study of follicular lymphoma, a subtype of indolent lymphoma, reported somewhat divergent genetic profiles in nodal and bone marrow lesions, although most mutations were common to both.^5^

Here, we examined time‐dependent and spatial ITH using autopsy samples in extranodal natural killer/T‐cell lymphoma, nasal type (ENKTL), a subtype of aggressive lymphoma.

**FIGURE 1:**
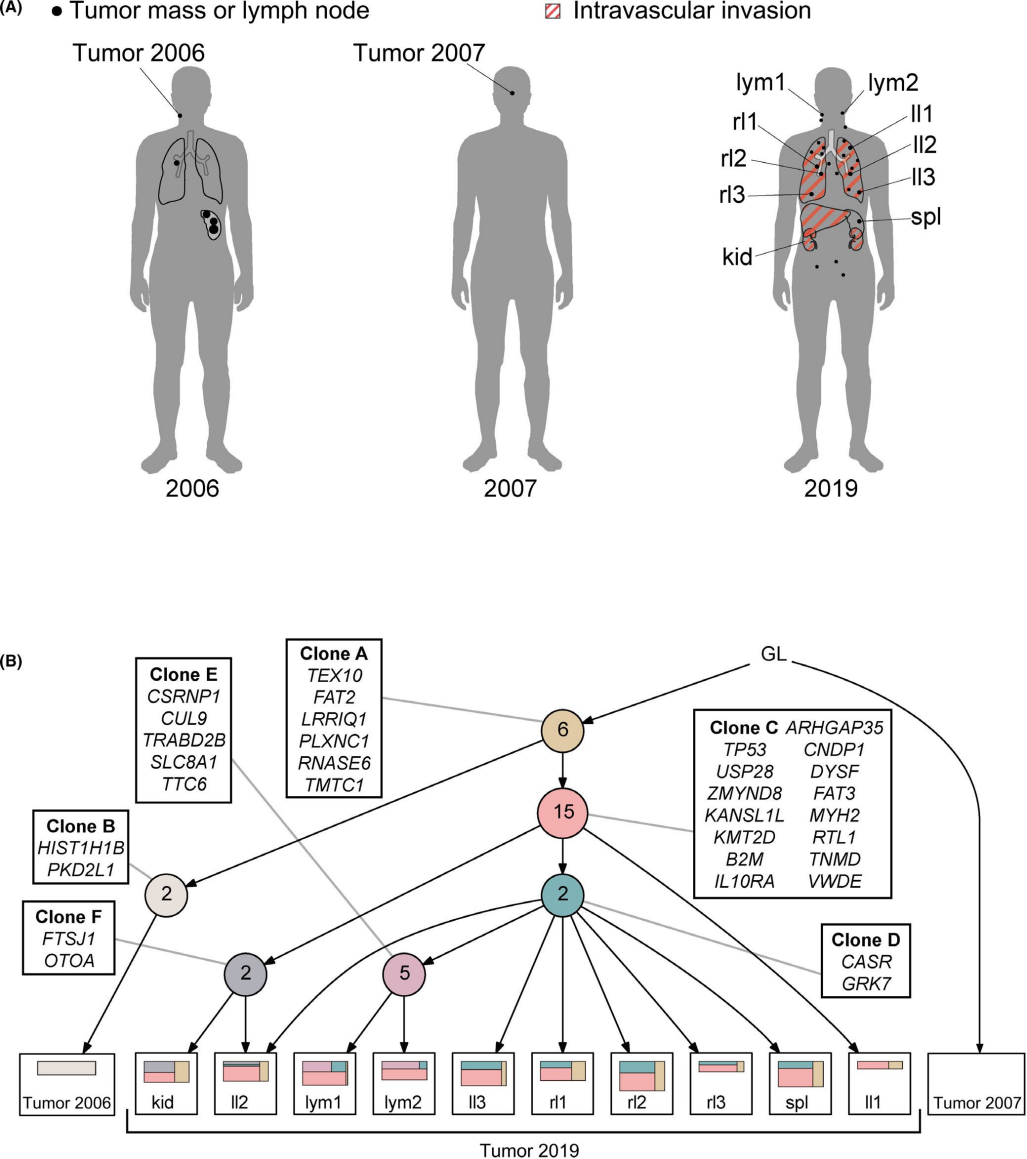
Phylogenetic tree indicating tumor evolution and intratumor heterogeneity. A, Schematics indicating tumor distribution and sites of sample collection. Black dots indicate a tumor mass; organs with red diagonal lines represent intravascular tumor invasions. kid, kidney; ll, left lung; lym, lymph node; rl1, right lung; spl, spleen. B, Phylogenetic tree constructed based on the LICHeE algorithm. Each colored circle represents a node (cluster). Numbers inside circles indicate the number of representative mutations in that clone. Squares located at leaves of the tree indicate clones in individual samples: colored squares within a sample indicate prevalence of indicated clones. GL, germline

Whole‐exome sequencing (WES) was performed for 12 samples taken at diagnosis, first relapse, and autopsy (Figure 1A). Deep amplicon‐based sequencing was performed for mutations detected by WES in all samples. Somatic copy number alteration (SCNAs) analysis for the WES data was performed using CopywriteR^6^ and GISTIC 2.0.^7^ A phylogenetic tree of tumor regions was constructed using the LICHeE^8^ pipeline. The detailed method is provided in Appendix S1.

In the pathological evaluation (Figure S1), there were no morphological differences between tumor cells of each site. In lung and kidney samples, intravascular invasion of tumor cells was apparently observed. On the other hand, in lymph node and spleen samples, tumors accompanying necrosis were observed, while vascular infiltration was not recognized.

In total, 330 mutations in 41 genes and 283 SCNAs at mutational loci were identified. Detail of genetic profiles are described in Tables S1, S2 and Figure S2.

Next, we constructed a phylogenetic tree of tumor regions using LICHeE (Figure 1B). After integrating 32 gene mutations, we identified six subclones (clones A‐F). The sample at diagnosis in 2006 and those at the second relapse in 2019 were derived from founder clone A. The sample in 2006 defined as clone B acquired 2 additional mutations to clone A. All other clones identified in samples in 2019 were derived from clone C, with 15 additional mutations based on clone A. Through branching evolution from clone C, clones D and F each harbored two additional mutations. Clone D further evolved into clone E by branching evolution. Remarkably, samples from the left lung (ll) exhibited various combinations: as noted above, ll1 had only two clones (clones A and C), while ll2 had four (clones A, C, D, and F) and ll3 had three (clones A, C, and D). Of note, clones D and F both developed through branched evolution from clone C. In contrast, all three samples from right lung (rl1‐3) consisted mainly of clone C and their clonal composition was similar. Comparable clonal composition was also observed in spleen (spl). Clone E was seen only in lymph nodes samples (lym1 and lym2) with three ancestral clones, resulting in four clones in total (clones A, C, D, and E). Clonal composition of the kidney (kid) sample was similar to that of ll2, consisting of clones A, C, and F. By contrast, none of these clones were identified in the sample taken in 2007.

In our study, it was remarkable that spatial ITH was relatively apparent in ll1‐3 and consisted of multiple clones. In particular, ll1 consisted of ancestral clones, suggesting that tumor cells in ll1 were resistant to first‐ and second‐line treatment and may underlie subsequent systemic relapse. Samples ll2 and ll3 might also have grown independently following emergence of new clones. By contrast, the clonal structure of rl1‐3 and samples from other organs was almost homogeneous, reflecting rapid disease progression from the time of second relapse to the time of death 2 months later. In pathological evaluation, we recognized that there were two types of tumor infiltration patterns: tumor formation and vascular infiltration. However, these findings were not associated with our genetic findings.

In conclusion, this analysis shows a clear phylogenic tree of aggressive lymphoma, confirming that dynamic time‐dependent and spatial ITH occurs even in systemic disease.

## Supporting information

Fig S1

Fig S2

Table S1

Table S2

Appendix S1
