# Supplementary figures and images for: Intratumor heterogeneity of lymphoma identified by multiregion sequencing of autopsy samples
Source: Cancer Sci. 2021 Nov 21;113(1):362–4. doi: 10.1111/cas.15178 (PMC8748235; doi:10.1111/cas.15178)

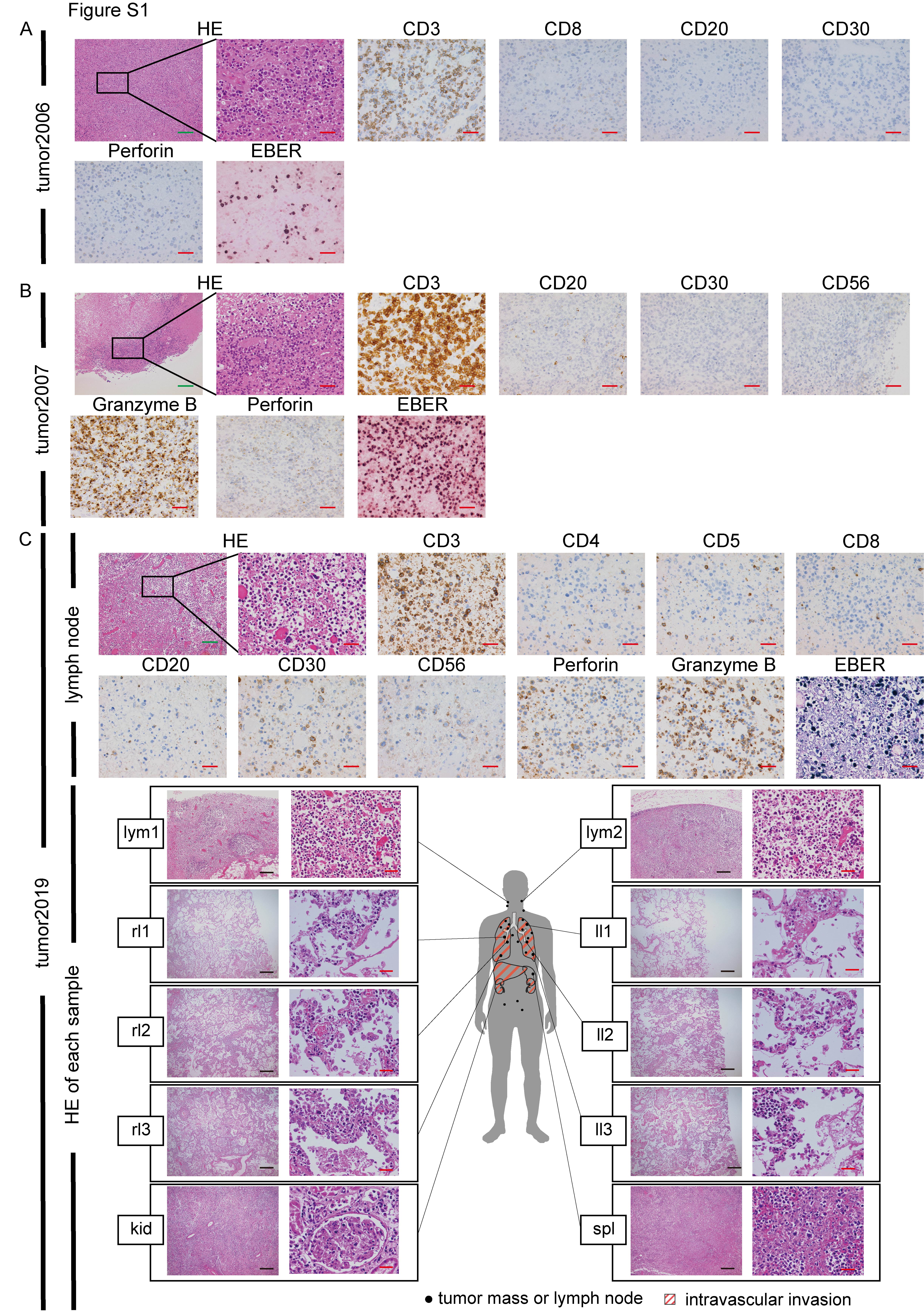

Supplement: Supplementary file 1 — Fig S1 [file CAS-113-362-s003.jpg]

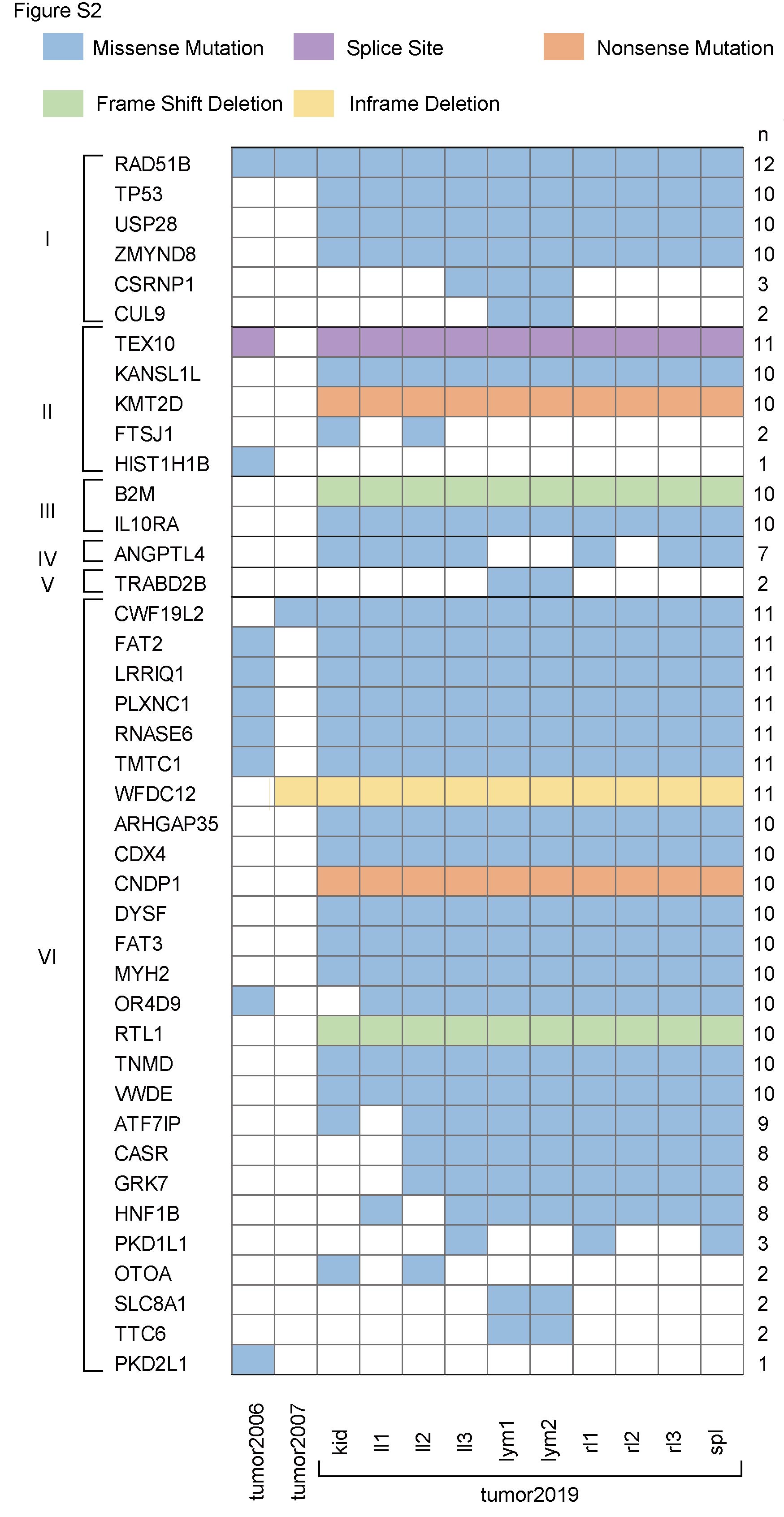

Supplement: Supplementary file 2 — Fig S2 [file CAS-113-362-s002.jpg]
