# Supplementary material for: Intratumor heterogeneity of lymphoma identified by multiregion sequencing of autopsy samples
Source: Cancer Sci. 2021 Nov 21;113(1):362–4. doi: 10.1111/cas.15178 (PMC8748235; doi:10.1111/cas.15178)
Supplement: Supplementary file 3 — Table S1 [file CAS-113-362-s004.pdf]

Table S1. Mutated genes in whole exome sequencing and amplicon seqencing analysis

Annotated Gene	Mutation Type	RefSeq_Amino	Acid Change	Chr	Position	Allele Change	Sample	VAF of WES[a]	VAF of deep amplicon sequencing
ANGPTL4	Missense_Mutation	NM_139314	p.G8E	Chr19	8429228	G>A	kid	0.17	0.123
ANGPTL4	Missense_Mutation	NM_139314	p.G8E	Chr19	8429228	G>A	ll2	---	0.086
ANGPTL4	Missense_Mutation	NM_139314	p.G8E	Chr19	8429228	G>A	ll3	---	0.055
ANGPTL4	Missense_Mutation	NM_139314	p.G8E	Chr19	8429228	G>A	rl3	---	0.028
ANGPTL4	Missense_Mutation	NM_139314	p.G8E	Chr19	8429228	G>A	spl	---	0.056
ANGPTL4	Missense_Mutation	NM_004491	p.V519F	Chr19	47423487	G>T	ll1	---	0.0245[b]
ANGPTL4	Missense_Mutation	NM_004491	p.V519F	Chr19	47423487	G>T	rl1	---	0.019[b]
ARHGAP35	Missense_Mutation	NM_004491	p.V519F	Chr19	47423487	G>T	kid	0.224	0.201
ARHGAP35	Missense_Mutation	NM_004491	p.V519F	Chr19	47423487	G>T	ll1	0.163	0.085
ARHGAP35	Missense_Mutation	NM_004491	p.V519F	Chr19	47423487	G>T	ll2	0.144	0.234
ARHGAP35	Missense_Mutation	NM_004491	p.V519F	Chr19	47423487	G>T	ll3	0.282	0.263
ARHGAP35	Missense_Mutation	NM_004491	p.V519F	Chr19	47423487	G>T	lym1	0.426	0.193
ARHGAP35	Missense_Mutation	NM_004491	p.V519F	Chr19	47423487	G>T	lym2	0.326	0.285
ARHGAP35	Missense_Mutation	NM_004491	p.V519F	Chr19	47423487	G>T	rl1	0.231	0.228
ARHGAP35	Missense_Mutation	NM_004491	p.V519F	Chr19	47423487	G>T	rl2	0.257	0.298
ARHGAP35	Missense_Mutation	NM_004491	p.V519F	Chr19	47423487	G>T	rl3	0.25	0.128
ARHGAP35	Missense_Mutation	NM_004491	p.V519F	Chr19	47423487	G>T	spl	0.236	0.277
ATF7IP	Missense_Mutation	NM_018179	p.G503D	Chr12	14578357	G>A	kid	0.28	0.342
ATF7IP	Missense_Mutation	NM_018179	p.G503D	Chr12	14578357	G>A	ll2	0.149	0.225
ATF7IP	Missense_Mutation	NM_018179	p.G503D	Chr12	14578357	G>A	ll3	0.226	0.390
ATF7IP	Missense_Mutation	NM_018179	p.G503D	Chr12	14578357	G>A	lym1	0.3	0.223
ATF7IP	Missense_Mutation	NM_018179	p.G503D	Chr12	14578357	G>A	lym2	0.214	0.191
ATF7IP	Missense_Mutation	NM_018179	p.G503D	Chr12	14578357	G>A	rl1	0.122	0.117
ATF7IP	Missense_Mutation	NM_018179	p.G503D	Chr12	14578357	G>A	rl2	0.111	0.314
ATF7IP	Missense_Mutation	NM_018179	p.G503D	Chr12	14578357	G>A	rl3	0.225	0.029
ATF7IP	Missense_Mutation	NM_018179	p.G503D	Chr12	14578357	G>A	spl	0.148	0.289
B2M	Frame_Shift_Del	NM_004048	p.L13fs	Chr15	45003781	CT>-	kid	0.096	0.074
B2M	Frame_Shift_Del	NM_004048	p.L13fs	Chr15	45003781	CT>-	ll1	0.103	0.033
B2M	Frame_Shift_Del	NM_004048	p.L13fs	Chr15	45003781	CT>-	ll2	0.11	0.074
B2M	Frame_Shift_Del	NM_004048	p.L13fs	Chr15	45003781	CT>-	ll3	0.313	0.249
B2M	Frame_Shift_Del	NM_004048	p.L13fs	Chr15	45003781	CT>-	lym1	0.38	0.389
B2M	Frame_Shift_Del	NM_004048	p.L13fs	Chr15	45003781	CT>-	lym2	0.359	0.255
B2M	Frame_Shift_Del	NM_004048	p.L13fs	Chr15	45003781	CT>-	rl1	0.243	0.153
B2M	Frame_Shift_Del	NM_004048	p.L13fs	Chr15	45003781	CT>-	rl2	0.226	0.340
B2M	Frame_Shift_Del	NM_004048	p.L13fs	Chr15	45003781	CT>-	rl3	0.247	0.073
B2M	Frame_Shift_Del	NM_004048	p.L13fs	Chr15	45003781	CT>-	spl	0.261	0.172
CASR	Missense_Mutation	NM_000388	p.T263M	Chr3	121980670	C>T	ll2	0.036	0.023
CASR	Missense_Mutation	NM_000388	p.T263M	Chr3	121980670	C>T	ll3	0.16	0.157
CASR	Missense_Mutation	NM_000388	p.T263M	Chr3	121980670	C>T	lym1	0.211	0.232
CASR	Missense_Mutation	NM_000388	p.T263M	Chr3	121980670	C>T	lym2	0.126	0.157
CASR	Missense_Mutation	NM_000388	p.T263M	Chr3	121980670	C>T	rl1	0.109	0.101
CASR	Missense_Mutation	NM_000388	p.T263M	Chr3	121980670	C>T	rl2	0.163	0.187
CASR	Missense_Mutation	NM_000388	p.T263M	Chr3	121980670	C>T	rl3	0.049	0.087
CASR	Missense_Mutation	NM_000388	p.T263M	Chr3	121980670	C>T	spl	0.184	0.099
CDX4	Missense_Mutation	NM_005193	p.A143T	ChrX	72667516	G>A	kid	0.311	0.538
CDX4	Missense_Mutation	NM_005193	p.A143T	ChrX	72667516	G>A	ll1	0.224	0.195
CDX4	Missense_Mutation	NM_005193	p.A143T	ChrX	72667516	G>A	ll2	0.368	0.503
CDX4	Missense_Mutation	NM_005193	p.A143T	ChrX	72667516	G>A	ll3	0.644	0.663
CDX4	Missense_Mutation	NM_005193	p.A143T	ChrX	72667516	G>A	lym1	0.681	0.734
CDX4	Missense_Mutation	NM_005193	p.A143T	ChrX	72667516	G>A	lym2	0.635	0.597
CDX4	Missense_Mutation	NM_005193	p.A143T	ChrX	72667516	G>A	rl1	0.491	0.442
CDX4	Missense_Mutation	NM_005193	p.A143T	ChrX	72667516	G>A	rl2	0.431	0.631
CDX4	Missense_Mutation	NM_005193	p.A143T	ChrX	72667516	G>A	rl3	0.556	0.294
CDX4	Missense_Mutation	NM_005193	p.A143T	ChrX	72667516	G>A	spl	0.463	0.576
CNDP1	Nonsense_Mutation	NM_032649	p.W173X	Chr18	72229333	G>A	kid	---	0.131
CNDP1	Nonsense_Mutation	NM_032649	p.W173X	Chr18	72229333	G>A	ll1	0.1	0.072
CNDP1	Nonsense_Mutation	NM_032649	p.W173X	Chr18	72229333	G>A	ll2	0.076	0.178
CNDP1	Nonsense_Mutation	NM_032649	p.W173X	Chr18	72229333	G>A	ll3	0.24	0.338
CNDP1	Nonsense_Mutation	NM_032649	p.W173X	Chr18	72229333	G>A	lym1	0.395	0.354
CNDP1	Nonsense_Mutation	NM_032649	p.W173X	Chr18	72229333	G>A	lym2	0.254	0.292
CNDP1	Nonsense_Mutation	NM_032649	p.W173X	Chr18	72229333	G>A	rl1	0.256	0.201
CNDP1	Nonsense_Mutation	NM_032649	p.W173X	Chr18	72229333	G>A	rl2	0.205	0.336
CNDP1	Nonsense_Mutation	NM_032649	p.W173X	Chr18	72229333	G>A	rl3	0.143	0.101
CNDP1	Nonsense_Mutation	NM_032649	p.W173X	Chr18	72229333	G>A	spl	0.371	0.282
CSRNP1	Missense_Mutation	NM_033027	p.R78Q	Chr3	39186720	C>T	ll3	0.13	0.016
CSRNP1	Missense_Mutation	NM_033027	p.R78Q	Chr3	39186720	C>T	lym1	0.114	0.128
CSRNP1	Missense_Mutation	NM_033027	p.R78Q	Chr3	39186720	C>T	lym2	0.094	0.141
CUL9	Missense_Mutation	NM_015089	p.T135A	Chr6	43152451	A>G	lym1	0.071	0.066
CUL9	Missense_Mutation	NM_015089	p.T135A	Chr6	43152451	A>G	lym2	0.101	0.078
CWF19L2	Missense_Mutation	NM_152434	p.A477P	Chr11	107299529	C>G	kid	0.129	0.170
CWF19L2	Missense_Mutation	NM_152434	p.A477P	Chr11	107299529	C>G	ll2	---	0.247
CWF19L2	Missense_Mutation	NM_152434	p.A477P	Chr11	107299529	C>G	ll3	0.294	0.398
CWF19L2	Missense_Mutation	NM_152434	p.A477P	Chr11	107299529	C>G	lym1	0.371	0.417
CWF19L2	Missense_Mutation	NM_152434	p.A477P	Chr11	107299529	C>G	lym2	0.273	0.343
CWF19L2	Missense_Mutation	NM_152434	p.A477P	Chr11	107299529	C>G	rl1	0.214	0.246
CWF19L2	Missense_Mutation	NM_152434	p.A477P	Chr11	107299529	C>G	rl2	0.31	0.368
CWF19L2	Missense_Mutation	NM_152434	p.A477P	Chr11	107299529	C>G	rl3	0.174	0.138
CWF19L2	Missense_Mutation	NM_152434	p.A477P	Chr11	107299529	C>G	spl	0.214	0.320
CWF19L2	Missense_Mutation	NM_152434	p.A477P	Chr11	107299529	C>G	tumor2007	---	0.463
CWF19L2	Missense_Mutation	NM_152434	p.A477P	Chr11	107299529	C>G	ll1	---	0.051[b]
DYSF	Missense_Mutation	NM_001130987	p.A100S	Chr2	71730402	G>T	kid	---	0.139
DYSF	Missense_Mutation	NM_001130987	p.A100S	Chr2	71730402	G>T	ll2	---	0.122
DYSF	Missense_Mutation	NM_001130987	p.A100S	Chr2	71730402	G>T	ll3	0.413	0.363
DYSF	Missense_Mutation	NM_001130987	p.A100S	Chr2	71730402	G>T	lym1	0.469	0.422
DYSF	Missense_Mutation	NM_001130987	p.A100S	Chr2	71730402	G>T	lym2	0.286	0.352
DYSF	Missense_Mutation	NM_001130987	p.A100S	Chr2	71730402	G>T	rl1	0.196	0.227
DYSF	Missense_Mutation	NM_001130987	p.A100S	Chr2	71730402	G>T	rl2	0.186	0.362
DYSF	Missense_Mutation	NM_001130987	p.A100S	Chr2	71730402	G>T	rl3	0.161	0.129
DYSF	Missense_Mutation	NM_001130987	p.A100S	Chr2	71730402	G>T	spl	0.16	0.276
DYSF	Missense_Mutation	NM_001130987	p.A100S	Chr2	71730402	G>T	ll1	---	0.05[b]
FAT2	Missense_Mutation	NM_001447	p.H3621Y	Chr5	150901293	G>A	kid	0.199	0.265
FAT2	Missense_Mutation	NM_001447	p.H3621Y	Chr5	150901293	G>A	ll1	0.191	0.083
FAT2	Missense_Mutation	NM_001447	p.H3621Y	Chr5	150901293	G>A	ll2	0.158	0.275
FAT2	Missense_Mutation	NM_001447	p.H3621Y	Chr5	150901293	G>A	ll3	0.41	0.407
FAT2	Missense_Mutation	NM_001447	p.H3621Y	Chr5	150901293	G>A	lym1	0.385	0.425
FAT2	Missense_Mutation	NM_001447	p.H3621Y	Chr5	150901293	G>A	lym2	0.314	0.339
FAT2	Missense_Mutation	NM_001447	p.H3621Y	Chr5	150901293	G>A	rl1	0.302	0.227
FAT2	Missense_Mutation	NM_001447	p.H3621Y	Chr5	150901293	G>A	rl2	0.24	0.377
FAT2	Missense_Mutation	NM_001447	p.H3621Y	Chr5	150901293	G>A	rl3	0.243	0.130
FAT2	Missense_Mutation	NM_001447	p.H3621Y	Chr5	150901293	G>A	spl	0.256	0.331
FAT2	Missense_Mutation	NM_001447	p.H3621Y	Chr5	150901293	G>A	tumor2006	0.064	0.063

FAT3	Missense_Mutation	NM_001008781	p.I4273T	Chr11	92616440	T>C	kid	0.129	0.141
FAT3	Missense_Mutation	NM_001008781	p.I4273T	Chr11	92616440	T>C	ll2	0.057	0.131
FAT3	Missense_Mutation	NM_001008781	p.I4273T	Chr11	92616440	T>C	ll3	0.162	0.194
FAT3	Missense_Mutation	NM_001008781	p.I4273T	Chr11	92616440	T>C	lym1	0.234	0.200
FAT3	Missense_Mutation	NM_001008781	p.I4273T	Chr11	92616440	T>C	lym2	0.175	0.169
FAT3	Missense_Mutation	NM_001008781	p.I4273T	Chr11	92616440	T>C	rl1	0.135	0.119
FAT3	Missense_Mutation	NM_001008781	p.I4273T	Chr11	92616440	T>C	rl2	0.066	0.182
FAT3	Missense_Mutation	NM_001008781	p.I4273T	Chr11	92616440	T>C	rl3	0.158	0.069
FAT3	Missense_Mutation	NM_001008781	p.I4273T	Chr11	92616440	T>C	spl	0.121	0.169
FAT3	Missense_Mutation	NM_001008781	p.I4273T	Chr11	92616440	T>C	ll1	---	0.041[b]
FTSJ1	Missense_Mutation	NM_012280	p.R44Q	ChrX	48336846	G>A	kid	0.136	0.155
FTSJ1	Missense_Mutation	NM_012280	p.R44Q	ChrX	48336846	G>A	ll2	---	0.039
GRK7	Missense_Mutation	NM_139209	p.R49C	Chr3	141497271	C>T	ll3	0.1	0.156
GRK7	Missense_Mutation	NM_139209	p.R49C	Chr3	141497271	C>T	lym1	0.183	0.177
GRK7	Missense_Mutation	NM_139209	p.R49C	Chr3	141497271	C>T	lym2	0.135	0.160
GRK7	Missense_Mutation	NM_139209	p.R49C	Chr3	141497271	C>T	rl1	---	0.089
GRK7	Missense_Mutation	NM_139209	p.R49C	Chr3	141497271	C>T	rl2	---	0.180
GRK7	Missense_Mutation	NM_139209	p.R49C	Chr3	141497271	C>T	spl	0.138	0.136
GRK7	Missense_Mutation	NM_139209	p.R49C	Chr3	141497271	C>T	ll2	---	0.012*
GRK7	Missense_Mutation	NM_139209	p.R49C	Chr3	141497271	C>T	rl3	---	0.022*
HIST1H1B	Missense_Mutation	NM_005322	p.P41T	Chr6	27835187	G>T	tumor2006	0.156	0.231
HNF1B	Missense_Mutation	NM_000458	p.R233C	Chr17	36093662	G>A	ll3	0.11	0.153
HNF1B	Missense_Mutation	NM_000458	p.R233C	Chr17	36093662	G>A	lym1	0.178	0.142
HNF1B	Missense_Mutation	NM_000458	p.R233C	Chr17	36093662	G>A	lym2	0.111	0.120
HNF1B	Missense_Mutation	NM_000458	p.R233C	Chr17	36093662	G>A	rl1	---	0.092
HNF1B	Missense_Mutation	NM_000458	p.R233C	Chr17	36093662	G>A	rl2	---	0.173
HNF1B	Missense_Mutation	NM_000458	p.R233C	Chr17	36093662	G>A	rl3	0.106	0.050
HNF1B	Missense_Mutation	NM_000458	p.R233C	Chr17	36093662	G>A	spl	0.098	0.095
HNF1B	Missense_Mutation	NM_000458	p.R233C	Chr17	36093662	G>A	ll1	---	0.026*
IL10RA	Missense_Mutation	NM_001558	p.N131D	Chr11	117863979	A>G	kid	0.356	0.289
IL10RA	Missense_Mutation	NM_001558	p.N131D	Chr11	117863979	A>G	ll2	0.196	0.255
IL10RA	Missense_Mutation	NM_001558	p.N131D	Chr11	117863979	A>G	ll3	0.48	0.360
IL10RA	Missense_Mutation	NM_001558	p.N131D	Chr11	117863979	A>G	lym1	0.423	0.407
IL10RA	Missense_Mutation	NM_001558	p.N131D	Chr11	117863979	A>G	lym2	0.354	0.330
IL10RA	Missense_Mutation	NM_001558	p.N131D	Chr11	117863979	A>G	rl1	0.241	0.248
IL10RA	Missense_Mutation	NM_001558	p.N131D	Chr11	117863979	A>G	rl2	0.174	0.367
IL10RA	Missense_Mutation	NM_001558	p.N131D	Chr11	117863979	A>G	rl3	0.234	0.145
IL10RA	Missense_Mutation	NM_001558	p.N131D	Chr11	117863979	A>G	spl	0.254	0.302
IL10RA	Missense_Mutation	NM_001558	p.N131D	Chr11	117863979	A>G	ll1	---	0.081*
KAMSL1L	Missense_Mutation	NM_152519	p.S738R	Chr2	210894584	G>C	ll1	---	0.045*
KANSL1L	Missense_Mutation	NM_152519	p.S738R	Chr2	210894584	G>C	kid	0.206	0.212
KANSL1L	Missense_Mutation	NM_152519	p.S738R	Chr2	210894584	G>C	ll2	0.122	0.268
KANSL1L	Missense_Mutation	NM_152519	p.S738R	Chr2	210894584	G>C	ll3	---	0.252
KANSL1L	Missense_Mutation	NM_152519	p.S738R	Chr2	210894584	G>C	lym1	---	0.218
KANSL1L	Missense_Mutation	NM_152519	p.S738R	Chr2	210894584	G>C	lym2	---	0.161
KANSL1L	Missense_Mutation	NM_152519	p.S738R	Chr2	210894584	G>C	rl1	0.211	0.117
KANSL1L	Missense_Mutation	NM_152519	p.S738R	Chr2	210894584	G>C	rl2	---	0.182
KANSL1L	Missense_Mutation	NM_152519	p.S738R	Chr2	210894584	G>C	rl3	0.184	0.106
KANSL1L	Missense_Mutation	NM_152519	p.S738R	Chr2	210894584	G>C	spl	---	0.213
KMT2D	Nonsense_Mutation	NM_003482	p.Q3745X	Chr12	49427255	G>A	kid	0.123	0.159
KMT2D	Nonsense_Mutation	NM_003482	p.Q3745X	Chr12	49427255	G>A	ll1	0.102	0.052
KMT2D	Nonsense_Mutation	NM_003482	p.Q3745X	Chr12	49427255	G>A	ll2	0.101	0.152
KMT2D	Nonsense_Mutation	NM_003482	p.Q3745X	Chr12	49427255	G>A	ll3	0.274	0.251
KMT2D	Nonsense_Mutation	NM_003482	p.Q3745X	Chr12	49427255	G>A	lym1	0.286	0.339
KMT2D	Nonsense_Mutation	NM_003482	p.Q3745X	Chr12	49427255	G>A	lym2	0.293	0.291
KMT2D	Nonsense_Mutation	NM_003482	p.Q3745X	Chr12	49427255	G>A	rl1	0.179	0.123
KMT2D	Nonsense_Mutation	NM_003482	p.Q3745X	Chr12	49427255	G>A	rl2	0.119	0.219
KMT2D	Nonsense_Mutation	NM_003482	p.Q3745X	Chr12	49427255	G>A	rl3	0.084	0.087
KMT2D	Nonsense_Mutation	NM_003482	p.Q3745X	Chr12	49427255	G>A	spl	0.154	0.199
LRRIQ1	Missense_Mutation	NM_001079910	p.H409N	Chr12	85449796	C>A	kid	0.143	0.217
LRRIQ1	Missense_Mutation	NM_001079910	p.H409N	Chr12	85449796	C>A	ll2	0.128	0.263
LRRIQ1	Missense_Mutation	NM_001079910	p.H409N	Chr12	85449796	C>A	ll3	0.377	0.386
LRRIQ1	Missense_Mutation	NM_001079910	p.H409N	Chr12	85449796	C>A	lym1	0.231	0.370
LRRIQ1	Missense_Mutation	NM_001079910	p.H409N	Chr12	85449796	C>A	lym2	0.333	0.262
LRRIQ1	Missense_Mutation	NM_001079910	p.H409N	Chr12	85449796	C>A	rl1	0.125	0.220
LRRIQ1	Missense_Mutation	NM_001079910	p.H409N	Chr12	85449796	C>A	rl2	0.282	0.337
LRRIQ1	Missense_Mutation	NM_001079910	p.H409N	Chr12	85449796	C>A	rl3	0.321	0.122
LRRIQ1	Missense_Mutation	NM_001079910	p.H409N	Chr12	85449796	C>A	spl	0.148	0.312
LRRIQ1	Missense_Mutation	NM_001079910	p.H409N	Chr12	85449796	C>A	tumor2006	---	0.111
LRRIQ1	Missense_Mutation	NM_001079910	p.H409N	Chr12	85449796	C>A	ll1	---	0.063*
MYH2	Missense_Mutation	NM_017534	p.V192A	Chr17	10447292	A>G	kid	0.198	0.225
MYH2	Missense_Mutation	NM_017534	p.V192A	Chr17	10447292	A>G	ll1	0.202	0.096
MYH2	Missense_Mutation	NM_017534	p.V192A	Chr17	10447292	A>G	ll2	0.151	0.255
MYH2	Missense_Mutation	NM_017534	p.V192A	Chr17	10447292	A>G	ll3	0.361	0.389
MYH2	Missense_Mutation	NM_017534	p.V192A	Chr17	10447292	A>G	lym1	0.423	0.378
MYH2	Missense_Mutation	NM_017534	p.V192A	Chr17	10447292	A>G	lym2	0.375	0.299
MYH2	Missense_Mutation	NM_017534	p.V192A	Chr17	10447292	A>G	rl1	0.327	0.262
MYH2	Missense_Mutation	NM_017534	p.V192A	Chr17	10447292	A>G	rl2	0.308	0.381
MYH2	Missense_Mutation	NM_017534	p.V192A	Chr17	10447292	A>G	rl3	0.286	0.160
MYH2	Missense_Mutation	NM_017534	p.V192A	Chr17	10447292	A>G	spl	0.295	0.342
OR4D9	Missense_Mutation	NM_001004711	p.G109E	Chr11	59282711	G>A	ll2	---	0.020
OR4D9	Missense_Mutation	NM_001004711	p.G109E	Chr11	59282711	G>A	ll3	0.04	0.140
OR4D9	Missense_Mutation	NM_001004711	p.G109E	Chr11	59282711	G>A	lym1	0.086	0.132
OR4D9	Missense_Mutation	NM_001004711	p.G109E	Chr11	59282711	G>A	lym2	---	0.100
OR4D9	Missense_Mutation	NM_001004711	p.G109E	Chr11	59282711	G>A	rl1	0.041	0.081
OR4D9	Missense_Mutation	NM_001004711	p.G109E	Chr11	59282711	G>A	rl2	0.083	0.187
OR4D9	Missense_Mutation	NM_001004711	p.G109E	Chr11	59282711	G>A	rl3	0.069	0.057
OR4D9	Missense_Mutation	NM_001004711	p.G109E	Chr11	59282711	G>A	spl	0.116	0.142
OR4D9	Missense_Mutation	NM_001004711	p.G109E	Chr11	59282711	G>A	tumor2006	---	0.042
OR4DP	Missense_Mutation	NM_001004711	p.G109E	Chr11	59282711	G>A	ll1	---	0.017*
OTOA	Missense_Mutation	NM_144672	p.R467C	Chr16	21726384	C>T	kid	0.099	0.095
OTOA	Missense_Mutation	NM_144672	p.R467C	Chr16	21726384	C>T	ll2	---	0.023
PKD1L1	Missense_Mutation	NM_138295	p.I760M	Chr7	47933648	A>C	ll3	---	0.102
PKD1L1	Missense_Mutation	NM_138295	p.I760M	Chr7	47933648	A>C	rl1	0.053	0.058
PKD1L1	Missense_Mutation	NM_138295	p.I760M	Chr7	47933648	A>C	spl	---	0.062
PKD2L1	Missense_Mutation	NM_138295	p.Q178K	Chr10	102058518	G>T	tumor2006	0.122	0.132
PLXNC1	Missense_Mutation	NM_005761	p.V571A	Chr12	94618013	T>C	kid	---	0.257
PLXNC1	Missense_Mutation	NM_005761	p.V571A	Chr12	94618013	T>C	ll2	0.209	0.265
PLXNC1	Missense_Mutation	NM_005761	p.V571A	Chr12	94618013	T>C	ll3	0.302	0.383
PLXNC1	Missense_Mutation	NM_005761	p.V571A	Chr12	94618013	T>C	lym1	0.368	0.426
PLXNC1	Missense_Mutation	NM_005761	p.V571A	Chr12	94618013	T>C	lym2	0.355	0.293
PLXNC1	Missense_Mutation	NM_005761	p.V571A	Chr12	94618013	T>C	rl1	0.214	0.234
PLXNC1	Missense_Mutation	NM_005761	p.V571A	Chr12	94618013	T>C	rl2	0.226	0.374

PLXNC1	Missense_Mutation	NM_005761	p.V571A	Chr12	94618013	T>C	rl3	0.296	0.156
PLXNC1	Missense_Mutation	NM_005761	p.V571A	Chr12	94618013	T>C	spl	0.2	0.317
PLXNC1	Missense_Mutation	NM_005761	p.V571A	Chr12	94618013	T>C	tumor2006	0.135	0.138
PLXNC1	Missense_Mutation	NM_005761	p.V571A	Chr12	94618013	T>C	ll1	---	0.082*
RAD51B	Missense_Mutation	NM_002877	p.R307Q	Chr14	68878207	G>A	kid	0.407	0.581
RAD51B	Missense_Mutation	NM_002877	p.R307Q	Chr14	68878207	G>A	ll1	0.556	0.526
RAD51B	Missense_Mutation	NM_002877	p.R307Q	Chr14	68878207	G>A	ll2	0.463	0.573
RAD51B	Missense_Mutation	NM_002877	p.R307Q	Chr14	68878207	G>A	ll3	0.58	0.609
RAD51B	Missense_Mutation	NM_002877	p.R307Q	Chr14	68878207	G>A	lym1	0.571	0.612
RAD51B	Missense_Mutation	NM_002877	p.R307Q	Chr14	68878207	G>A	lym2	0.458	0.596
RAD51B	Missense_Mutation	NM_002877	p.R307Q	Chr14	68878207	G>A	rl1	0.456	0.555
RAD51B	Missense_Mutation	NM_002877	p.R307Q	Chr14	68878207	G>A	rl2	0.486	0.607
RAD51B	Missense_Mutation	NM_002877	p.R307Q	Chr14	68878207	G>A	rl3	0.548	0.557
RAD51B	Missense_Mutation	NM_002877	p.R307Q	Chr14	68878207	G>A	spl	0.447	0.595
RAD51B	Missense_Mutation	NM_002877	p.R307Q	Chr14	68878207	G>A	tumor2006	0.419	0.591
RAD51B	Missense_Mutation	NM_001321812	p.R307Q	Chr14	68878207	G>A	tumor2007	0.162	0.217
RNASE6	Missense_Mutation	NM_005615	p.G109R	Chr14	21250183	G>A	kid	0.182	0.205
RNASE6	Missense_Mutation	NM_005615	p.G109R	Chr14	21250183	G>A	ll1	0.187	0.076
RNASE6	Missense_Mutation	NM_005615	p.G109R	Chr14	21250183	G>A	ll2	0.225	0.242
RNASE6	Missense_Mutation	NM_005615	p.G109R	Chr14	21250183	G>A	ll3	0.407	0.348
RNASE6	Missense_Mutation	NM_005615	p.G109R	Chr14	21250183	G>A	lym1	0.424	0.391
RNASE6	Missense_Mutation	NM_005615	p.G109R	Chr14	21250183	G>A	lym2	0.328	0.337
RNASE6	Missense_Mutation	NM_005615	p.G109R	Chr14	21250183	G>A	rl1	0.208	0.223
RNASE6	Missense_Mutation	NM_005615	p.G109R	Chr14	21250183	G>A	rl2	0.19	0.345
RNASE6	Missense_Mutation	NM_005615	p.G109R	Chr14	21250183	G>A	rl3	0.284	0.135
RNASE6	Missense_Mutation	NM_005615	p.G109R	Chr14	21250183	G>A	spl	0.225	0.312
RNASE6	Missense_Mutation	NM_005615	p.G109R	Chr14	21250183	G>A	tumor2006	0.074	0.193
RTL1	Frame_Shift_Del	NM_001134888	p.L809fs	Chr14	101348700	AG>-	kid	0.099	0.082
RTL1	Frame_Shift_Del	NM_001134888	p.L809fs	Chr14	101348700	AG>-	ll1	0.037	0.023
RTL1	Frame_Shift_Del	NM_001134888	p.L809fs	Chr14	101348700	AG>-	ll2	0.068	0.084
RTL1	Frame_Shift_Del	NM_001134888	p.L809fs	Chr14	101348700	AG>-	ll3	0.177	0.149
RTL1	Frame_Shift_Del	NM_001134888	p.L809fs	Chr14	101348700	AG>-	lym1	0.231	0.157
RTL1	Frame_Shift_Del	NM_001134888	p.L809fs	Chr14	101348700	AG>-	lym2	0.079	0.115
RTL1	Frame_Shift_Del	NM_001134888	p.L809fs	Chr14	101348700	AG>-	rl1	0.115	0.061
RTL1	Frame_Shift_Del	NM_001134888	p.L809fs	Chr14	101348700	AG>-	rl2	0.094	0.124
RTL1	Frame_Shift_Del	NM_001134888	p.L809fs	Chr14	101348700	AG>-	rl3	0.124	0.044
RTL1	Frame_Shift_Del	NM_001134888	p.L809fs	Chr14	101348700	AG>-	spl	0.091	0.128
SLC8A1	Missense_Mutation	NM_021097	p.E474K	Chr2	40656001	C>T	lym1	0.05	0.067
SLC8A1	Missense_Mutation	NM_021097	p.E474K	Chr2	40656001	C>T	lym2	---	0.011*
TEX10	Splice_Site	NM_017746	---	Chr9	103102538	C>T	kid	---	0.378
TEX10	Splice_Site	NM_017746	---	Chr9	103102538	C>T	ll1	0.18	0.129
TEX10	Splice_Site	NM_017746	---	Chr9	103102538	C>T	ll2	0.196	0.360
TEX10	Splice_Site	NM_017746	---	Chr9	103102538	C>T	ll3	0.327	0.433
TEX10	Splice_Site	NM_017746	---	Chr9	103102538	C>T	lym1	0.353	0.453
TEX10	Splice_Site	NM_017746	---	Chr9	103102538	C>T	lym2	0.514	0.389
TEX10	Splice_Site	NM_017746	---	Chr9	103102538	C>T	rl1	0.244	0.283
TEX10	Splice_Site	NM_017746	---	Chr9	103102538	C>T	rl2	0.27	0.397
TEX10	Splice_Site	NM_017746	---	Chr9	103102538	C>T	rl3	0.429	0.178
TEX10	Splice_Site	NM_017746	---	Chr9	103102538	C>T	spl	0.31	0.373
TEX10	Splice_Site	NM_017746	---	Chr9	103102538	C>T	tumor2006	---	0.141
TMTC1	Missense_Mutation	NM_175861	p.L619S	Chr12	29669409	A>G	kid	0.2	0.234
TMTC1	Missense_Mutation	NM_175861	p.L619S	Chr12	29669409	A>G	ll2	0.228	0.221
TMTC1	Missense_Mutation	NM_175861	p.L619S	Chr12	29669409	A>G	ll3	0.327	0.352
TMTC1	Missense_Mutation	NM_175861	p.L619S	Chr12	29669409	A>G	lym1	0.353	0.450
TMTC1	Missense_Mutation	NM_175861	p.L619S	Chr12	29669409	A>G	lym2	0.275	0.284
TMTC1	Missense_Mutation	NM_175861	p.L619S	Chr12	29669409	A>G	rl1	0.189	0.232
TMTC1	Missense_Mutation	NM_175861	p.L619S	Chr12	29669409	A>G	rl2	0.215	0.396
TMTC1	Missense_Mutation	NM_175861	p.L619S	Chr12	29669409	A>G	rl3	0.23	0.153
TMTC1	Missense_Mutation	NM_175861	p.L619S	Chr12	29669409	A>G	spl	0.224	0.292
TMTC1	Missense_Mutation	NM_175861	p.L619S	Chr12	29669409	A>G	tumor2006	---	0.09[b]
TMTC1	Missense_Mutation	NM_175861	p.L619S	Chr12	29669409	A>G	ll1	---	0.088[b]
TNMD	Missense_Mutation	NM_022144	p.R301H	ChrX	99854662	G>A	kid	0.125	0.230
TNMD	Missense_Mutation	NM_022144	p.R301H	ChrX	99854662	G>A	ll1	0.122	0.044
TNMD	Missense_Mutation	NM_022144	p.R301H	ChrX	99854662	G>A	ll2	0.185	0.230
TNMD	Missense_Mutation	NM_022144	p.R301H	ChrX	99854662	G>A	ll3	0.179	0.327
TNMD	Missense_Mutation	NM_022144	p.R301H	ChrX	99854662	G>A	lym1	0.289	0.333
TNMD	Missense_Mutation	NM_022144	p.R301H	ChrX	99854662	G>A	lym2	0.294	0.277
TNMD	Missense_Mutation	NM_022144	p.R301H	ChrX	99854662	G>A	rl1	0.212	0.195
TNMD	Missense_Mutation	NM_022144	p.R301H	ChrX	99854662	G>A	rl2	0.237	0.312
TNMD	Missense_Mutation	NM_022144	p.R301H	ChrX	99854662	G>A	rl3	0.214	0.126
TNMD	Missense_Mutation	NM_022144	p.R301H	ChrX	99854662	G>A	spl	0.319	0.279
TP53	Missense_Mutation	NM_000546	p.H214R	Chr17	7578208	T>C	kid	0.151	0.295
TP53	Missense_Mutation	NM_000546	p.H214R	Chr17	7578208	T>C	ll1	0.148	0.113
TP53	Missense_Mutation	NM_000546	p.H214R	Chr17	7578208	T>C	ll2	0.172	0.326
TP53	Missense_Mutation	NM_000546	p.H214R	Chr17	7578208	T>C	ll3	0.423	0.550
TP53	Missense_Mutation	NM_000546	p.H214R	Chr17	7578208	T>C	lym1	0.513	0.591
TP53	Missense_Mutation	NM_000546	p.H214R	Chr17	7578208	T>C	lym2	0.532	0.432
TP53	Missense_Mutation	NM_000546	p.H214R	Chr17	7578208	T>C	rl1	0.306	0.288
TP53	Missense_Mutation	NM_000546	p.H214R	Chr17	7578208	T>C	rl2	0.203	0.545
TP53	Missense_Mutation	NM_000546	p.H214R	Chr17	7578208	T>C	rl3	0.493	0.164
TP53	Missense_Mutation	NM_000546	p.H214R	Chr17	7578208	T>C	spl	0.21	0.459
TRABD2B	Missense_Mutation	NM_001194986	p.D259G	Chr1	48267182	T>C	lym1	0.078	0.078
TRABD2B	Missense_Mutation	NM_001194986	p.D259G	Chr1	48267182	T>C	lym2	0.12	0.097
TTC6	Missense_Mutation	NM_001310135	p.L1491I	Chr14	38276633	C>A	lym1	---	0.093
TTC6	Missense_Mutation	NM_001310135	p.L1491I	Chr14	38276633	C>A	lym2	0.167	0.087
USP28	Missense_Mutation	NM_001346264	p.N462D	Chr11	113678780	T>C	kid	0.149	0.140
USP28	Missense_Mutation	NM_001346264	p.N462D	Chr11	113678780	T>C	ll2	---	0.165
USP28	Missense_Mutation	NM_001346264	p.N462D	Chr11	113678780	T>C	ll3	0.25	0.227
USP28	Missense_Mutation	NM_001346264	p.N462D	Chr11	113678780	T>C	lym1	0.194	0.229
USP28	Missense_Mutation	NM_001346264	p.N462D	Chr11	113678780	T>C	lym2	0.19	0.178
USP28	Missense_Mutation	NM_001346264	p.N462D	Chr11	113678780	T>C	rl2	0.144	0.203
USP28	Missense_Mutation	NM_001346264	p.N462D	Chr11	113678780	T>C	spl	0.16	0.184
USP28	Missense_Mutation	NM_001346264	p.N462D	Chr11	113678780	T>C	ll1	---	0.047[b]
USP28	Missense_Mutation	NM_001346264	p.N462D	Chr11	113678780	T>C	rl1	---	0.109[b]
USP28	Missense_Mutation	NM_001346264	p.N462D	Chr11	113678780	T>C	rl3	---	0.074[b]
VWDE	Missense_Mutation	NM_001135924	p.W1416R	Chr7	12381709	A>T	kid	0.232	0.251
VWDE	Missense_Mutation	NM_001135924	p.W1416R	Chr7	12381709	A>T	ll1	0.118	0.079
VWDE	Missense_Mutation	NM_001135924	p.W1416R	Chr7	12381709	A>T	ll2	0.118	0.258
VWDE	Missense_Mutation	NM_001135924	p.W1416R	Chr7	12381709	A>T	ll3	0.293	0.395
VWDE	Missense_Mutation	NM_001135924	p.W1416R	Chr7	12381709	A>T	lym1	0.338	0.403
VWDE	Missense_Mutation	NM_001135924	p.W1416R	Chr7	12381709	A>T	lym2	0.281	0.328
VWDE	Missense_Mutation	NM_001135924	p.W1416R	Chr7	12381709	A>T	rl1	0.213	0.236
VWDE	Missense_Mutation	NM_001135924	p.W1416R	Chr7	12381709	A>T	rl2	0.236	0.378

VWDE	Missense_Mutation	NM_001135924	p.W1416R	Chr7	12381709	A>T	rl3	0.284	0.146
VWDE	Missense_Mutation	NM_001135924	p.W1416R	Chr7	12381709	A>T	spl	0.247	0.357
WFDC12	In_Frame_Del	NM_080869	p.62_63del	Chr20	43752799	AAC>-	kid	0.174	0.121
WFDC12	In_Frame_Del	NM_080869	p.62_63del	Chr20	43752799	AAC>-	ll1	0.142	0.029
WFDC12	In_Frame_Del	NM_080869	p.62_63del	Chr20	43752799	AAC>-	ll2	0.142	0.126
WFDC12	In_Frame_Del	NM_080869	p.62_63del	Chr20	43752799	AAC>-	ll3	0.244	0.199
WFDC12	In_Frame_Del	NM_080869	p.62_63del	Chr20	43752799	AAC>-	lym1	0.317	0.231
WFDC12	In_Frame_Del	NM_080869	p.62_63del	Chr20	43752799	AAC>-	lym2	0.314	0.163
WFDC12	In_Frame_Del	NM_080869	p.62_63del	Chr20	43752799	AAC>-	rl1	0.149	0.113
WFDC12	In_Frame_Del	NM_080869	p.62_63del	Chr20	43752799	AAC>-	rl2	0.186	0.168
WFDC12	In_Frame_Del	NM_080869	p.62_63del	Chr20	43752799	AAC>-	rl3	0.23	0.067
WFDC12	In_Frame_Del	NM_080869	p.62_63del	Chr20	43752799	AAC>-	spl	0.173	0.191
WFDC12	In_Frame_Del	NM_080869	p.62_63del	Chr20	43752799	AAC>-	tumor2007	---	0.736
ZMYND8	Missense_Mutation	NM_183047	p.M1V	Chr20	45985414	T>C	kid	0.255	0.231
ZMYND8	Missense_Mutation	NM_183047	p.M1V	Chr20	45985414	T>C	ll1	0.148	0.077
ZMYND8	Missense_Mutation	NM_183047	p.M1V	Chr20	45985414	T>C	ll2	0.167	0.239
ZMYND8	Missense_Mutation	NM_183047	p.M1V	Chr20	45985414	T>C	ll3	0.236	0.346
ZMYND8	Missense_Mutation	NM_183047	p.M1V	Chr20	45985414	T>C	lym1	0.311	0.358
ZMYND8	Missense_Mutation	NM_183047	p.M1V	Chr20	45985414	T>C	lym2	0.205	0.292
ZMYND8	Missense_Mutation	NM_183047	p.M1V	Chr20	45985414	T>C	rl1	0.143	0.215
ZMYND8	Missense_Mutation	NM_183047	p.M1V	Chr20	45985414	T>C	rl2	0.177	0.319
ZMYND8	Missense_Mutation	NM_183047	p.M1V	Chr20	45985414	T>C	rl3	0.245	0.140
ZMYND8	Missense_Mutation	NM_183047	p.M1V	Chr20	45985414	T>C	spl	0.31	0.282

[a] We applied criteria of candidate mutations as previously described[1]: (i) Fisher's exact $P \leq 0.01$; (ii) ≥5 variant reads in tumor samples; (iii) a VAF in tumor samples ≥0.07; and (iv) a VAF in matched normal samples <0.07. We excluded (i) synonymous SNVs and (ii) known variants listed in the 1000 Genomes Project (East Asian population, October 2014 release). We excluded mapping errors using visual inspection with Integrative Genomics Viewer (IGV) 2.6.3 (https://www.broadinstitute.org/igv/).

[b] VAFs of deep amplicon sequencing were confirmed by visual inspection with IGV 2.6.3.

Chr, chromosome; VAF, variant allele frequency; WES, whole-exome sequencing; kid, kidney; spl, spleen; lym1, lymph node 1; lym2, lymph node 2; rl1, right lung 1; rl2, right lung 2; rl3, right lung 3; ll1: left lung 1; ll2, left lung 2; ll3, left lung 3.
