# Supplementary material for: Intratumor heterogeneity of lymphoma identified by multiregion sequencing of autopsy samples
Source: Cancer Sci. 2021 Nov 21;113(1):362–4. doi: 10.1111/cas.15178 (PMC8748235; doi:10.1111/cas.15178)
Supplement: Supplementary file 4 — Table S2 [file CAS-113-362-s001.pdf]

Table S2. Total copy number at mutant loci inferred from whole exome sequencing.

Gene	Cytoband	Total copy number											
		tumor2019										tumor2006	tumor2007
		kid	ll1	ll2	ll3	lym1	lym2	rl1	rl2	rl3	spl		
ANGPTL4	19p13.2	1	1	1	0	0	0	0	1	1	0	1	1
FTSJ1	Xp11.23	3	2	2	3	3	3	3	2	3	2	2	1
HIST1H1B	6p22.1	2	2	2	2	2	2	3	3	3	3	2	1
TEX10	9q31.1	2	2	3	3	4	3	3	3	3	3	2	2
IL10RA	11q23.3	1	2	1	1	1	0	1	1	1	1	2	2
B2M	15q21.1	2	3	2	3	3	2	3	3	3	3	2	2
ARHGAP35	19q13.32	1	1	1	1	1	0	1	1	1	1	1	4
ATF7IP	12p13.1	3	2	3	3	3	3	2	2	3	2	2	2
CASR	3q21.1	3	2	3	3	3	3	3	3	3	3	3	2
CDX4	Xq13.2	3	2	2	3	3	3	3	2	3	2	4	1
CNDP1	18q22.3	1	1	4	4	2	2	4	1	1	3	2	4
CWF19L2	11q22.3	1	2	1	1	1	1	1	2	1	1	2	2
DYSF	2p13.2	1	2	2	2	1	2	2	1	2	2	3	2
FAT2	5q33.1	1	2	1	2	1	1	2	2	2	2	2	2
FAT3	11q14.3	1	2	1	1	1	1	1	2	1	1	2	2
GRK7	3q23	3	2	3	3	3	3	3	3	3	3	3	2
HNF1B	17q12	1	1	1	2	1	1	1	1	2	1	1	1
LRRIQ1	12q21.31	2	2	2	3	3	3	2	2	2	2	2	2
MYH2	17p13.1	3	3	2	3	3	3	3	2	3	3	1	1
OR4D9	11q12.1	1	1	1	1	1	1	1	1	1	1	2	1
OTOA	16p12.2	1	1	1	1	1	0	1	1	1	1	1	1
PKD1L1	7p12.3	2	2	2	2	2	2	2	2	2	2	3	2
PKD2L1	10q24.31	2	2	2	2	1	2	2	2	2	2	3	2
PLXNC1	12q22	2	2	2	3	3	3	2	2	2	2	2	2
RNASE6	14q11.2	2	2	2	1	1	0	2	1	2	1	1	2
RTL1	14q32.2	2	2	2	2	2	2	2	2	2	2	2	2
SLC8A1	2p22.1	2	2	2	2	2	2	2	2	2	2	3	2
TMTC1	12p11.22	3	2	3	3	3	3	2	2	3	2	2	2
TNMD	Xq22.1	3	2	2	3	3	3	3	2	3	2	4	1
VWDE	7p21.3	2	2	2	2	2	2	2	2	2	2	2	2
WFDC12	20q13.12	2	2	2	2	3	3	2	2	2	2	2	1
CSRNP1	3p22.2	3	2	3	3	3	3	3	3	3	3	2	2
CUL9	6p21.1	2	2	3	2	2	2	3	3	3	3	2	1
RAD51B	14q24.1	2	2	2	2	2	2	2	2	2	2	2	2
TP53	17p13.1	1	1	1	1	0	0	1	1	1	0	1	1
USP28	11q23.2	1	2	1	1	1	0	1	2	1	1	2	2
ZMYND8	20q13.12	2	2	2	2	3	3	2	2	2	2	2	1

kid, kidney; spl, spleen; lym1, lymph node 1; lym2, lymph node 2; rl1, right lung 1; rl2, right lung 2; rl3, right lung 3; ll1: left lung 1; ll2, left lung 2; ll3, left lung 3.
