# Supplementary material for: Intratumor heterogeneity of lymphoma identified by multiregion sequencing of autopsy samples
Source: Cancer Sci. 2021 Nov 21;113(1):362–4. doi: 10.1111/cas.15178 (PMC8748235; doi:10.1111/cas.15178)
Supplement: Supplementary file 5 — Appendix S1 [file CAS-113-362-s005.docx]

**Appendix S1**

**Supplementary Information**

**METHODS**

**Disease history**

A 48-year-old man with no past medical history of cancer presented with cervical and hilar lymphadenopathy and splenomegaly in 2006. Based on pathological examination of a swollen cervical lymph node, he was diagnosed with peripheral T-cell lymphoma, not otherwise specified (PTCL, NOS). He received 8 cycles of CHOP (cyclophosphamide, doxorubicin, vincristine, and prednisone) and achieved complete remission (CR). After one year, he experienced nasal congestion and displayed a mass in the nasal cavity. From pathological examination of biopsied material from the nasal mass, he was diagnosed with Extranodal NK/T-cell lymphoma, nasal type (ENKTL). He received 45Gy radiotherapy and 6 cycles of DeVIC (dexamethasone, etoposide, ifosfamide, and carboplatin), and achieved CR. After 12 years, at an annual follow-up, he demonstrated multiple masses in both lungs and his condition rapidly deteriorated. The patient died 2 months later due to progressive disease. An autopsy was performed to determine distribution of lesions and cause of death.

Pathologically, swollen lymph nodes were infiltrated by ENKTL tumor cells that were EBV-encoded small RNAs (EBER)^+^, CD3^+^, Perforin^+^, GranzymeB^+^, CD56^+^, CD30^+^, CD4^-^, CD5^-^, and CD8^-^. Vascular structures of lung and kidney were massively infiltrated by tumor cells, which likely accounted for the patient’s death (Supplemental figure S1C). At autopsy, biopsy samples in 2006 and 2007 were re-assessed and each sample showed CD3^+^, Perforin^+^, and EBER^+^ and CD3^+^, Granzyme B^+^, Perforin^+^, EBER^+^ respectively. Both were re-diagnosed as ENKTL (Supplemental figure S1A, S1B).

**Patient samples**

Ten samples were collected at autopsy (two cervical lymph nodes, spleen, kidney, three right lung masses, and three left lung masses) and lymph node and nasal mass samples were collected for biopsy in 2006 and 2007, respectively. A sample was collected from the buccal mucosa as a normal reference.

**DNA extraction**

Genomic DNAs were extracted from fresh autopsy samples using QIAamp DNA Mini Kit (Qiagen) and from formalin-fixed paraffin-embedded (FFPE) samples using a Generead DNA FFPE Tissue Kit (Qiagen). The sample from buccal mucosa was extracted using a QIAamp DNA Mini Kit. Due to the small quantity of DNA in FFPE samples and in the buccal mucosa sample, these samples were subjected to whole genome amplification using the REPLI-g Mini Kit (Qiagen). All experiments were performed according to the manufacturer’s instructions.

**Whole exome sequencing (WES) and analysis**

WES libraries were constructed using Human All Exon v7 SureSelect XT kits (Agilent Technologies) according to the manufacturer’s instructions. Captured exome libraries were multiplexed and sequenced on the Illumina Hiseq XTen platform using the standard 150 bp paired-end protocol (Macrogen). We performed sequence alignment and mutation calling of WES using the Genomon2 pipeline (https://github.com/Genomon-Project). BWA alignments of reads were performed using the reference genome hg19. We applied criteria of candidate mutations as previously described^1^: (i) Fisher's exact *P* ≤ 0.01; (ii) ≥5 variant reads in tumor samples; (iii) a VAF in tumor samples ≥0.07; and (iv) a VAF in matched normal samples <0.07. We excluded (i) synonymous SNVs and (ii) known variants listed in the 1000 Genomes Project (East Asian population, October 2014 release). We excluded mapping errors using visual inspection with Integrative Genomics Viewer (IGV) 2.6.3 (https://www.broadinstitute.org/igv/).

**Copy number analysis**

We performed copy number analysis of WES data using CopywriteR (https://github.com/PeeperLab/CopywriteR)^2^. We identified significant focal CNVs for CopywriteR output using GISTIC 2.0 ([https://github.com/genepattern/GISTIC_2.0](about:blank))^3^.

**Deep target amplicon sequencing**

We performed deep target amplicon sequencing to validate WES analysis as previously described, with minor modification^4^. Briefly, we amplified DNA covering the 41 mutated sites detected by WES using PCR primers with a NOTI linker (*Online Supplementary Table S4*). All PCR products were pooled, purified using a QIAquick PCR Purification Kit (QIAGEN), and digested using NotI enzyme (Takara Bio). Digested PCR products were purified and then ligated with T4 DNA ligase (Takara Bio) for 24 hours. Ligated DNAs were sonicated using S220 Focused-ultrasonicator (Covaris), and then libraries were constructed using SureSelect XT HS and XT Low input kits (Agilent Technologies), according to a modified version of the manufacturer’s method. Target libraries were multiplexed and sequenced on the Illumina Hiseq XTen platform using a standard 150 bp paired-end protocol (Macrogen). We performed sequence alignment and mutation calling using the Genomon2 pipeline.

**Clonality analysis and phylogenies**

We used the Lichee pipeline (http://viq854.github.io/lichee)^5^ to construct a tumor phylogenetic tree using variant allele frequencies (VAFs) of mutations determined by deep target amplicon sequencing using these parameters: *-maxVAFAbsent 0.045 -minVAFPresent 0.005 -minRobustNodeSupport 3*.

**References**

1. K Kataoka, Y Nagata, A Kitanaka, et al. Integrated molecular analysis of adult T cell leukemia/lymphoma. Nat Genet. 2015;47(11):1304-15.
2. T Kuilman, A Velds, K Kemper, et al. CopywriteR: DNA copy number detection from off-target sequence data. Genome Biol. 2015;27(16):49.
3. CH Mermel, SE Schumacher, B Hill, et al. GISTIC2.0 facilitates sensitive and confident localization of the targets of focal somatic copy-number alteration in human cancers. Genome Biol. 2011;12(4):R41.
4. K Yoshida, M Sanada, Y Shiraishi, et al. Frequent pathway mutations of splicing machinery in myelodysplasia. Nature. 2011;478(7367):64-9.
5. V Popic, R Salari, I Hajirasouliha, et al. Fast and scalable inference of multi-sample cancer lineages. Genome Biol. 2015;16(1):91.

**Supplementary Figure legend**

**Supplementary Figure S1: Representative pathological images of tumor specimens.**

1. Images showing the biopsy sample taken from cervical lymph node from the patient in 2006. These images show the Hematoxylin Eosin (HE) staining and immunostaining of CD3, CD8, CD20, CD30, Perforin, and EBV-encoded small RNAs (EBER).
2. Images showing the biopsy sample taken from nasal cavity mass of the patient in 2007. These images show HE staining and immunostaining of CD3, CD20, CD30, CD56, Granzyme B, Perforin, and EBER.
3. Representative images for samples taken at autopsy of the patient in 2019. These images show HE staining and immunostaining of CD3, CD4, CD5, CD8, CD20, CD30, CD56, Perforin, Granzyme B, and EBER of cervical lymph node. And the lower half of figure shows the HE staining pictures of each sample. kid, kidney; spl, spleen; lym1, lymph node 1; lym2, lymph node 2; rl1, right lung 1; rl2, right lung 2; rl3, right lung 3; ll1: left lung 1; ll2, left lung 2; ll3, left lung 3. Black dots indicate a tumor mass; organs painted with red diagonal lines represent intravascular tumor invasions.

Original magnification: ×40 for left figures; ×400 for right figures; ×100 for the top figures of A, B, and C. Scale bars: black, 500µm; green, 200µm; red, 50µm.

**Supplementary Figure 2: Genetic profile.**

Distribution of somatic mutations across 12 samples are shown. At left, gene ontology is indicated: I, tumor suppressor; II, epigenetic factor; III, immune activity; IV, angiogenesis; V, Wnt signaling pathway; VI, others. The number of samples with mutations is shown at right (n). Colored cells indicate the presence of mutation, and colors correspond as follows to the type of mutation: blue, missense mutation; purple, splice site mutation; orange, nonsense mutation, green; frame shift deletion; yellow; inframe deletion.
